# The time course of physiological adaptations to high‐intensity interval training in older adults

**DOI:** 10.1002/agm2.12127

**Published:** 2020-09-17

**Authors:** Philip J. J. Herrod, James E. M. Blackwell, Catherine L. Boereboom, Philip J. Atherton, John P. Williams, Jonathan N. Lund, Bethan E. Phillips

**Affiliations:** ^1^ Medical Research Council‐Versus Arthritis Centre for Musculoskeletal Ageing Research NIHR Nottingham Biomedical Research Centre Royal Derby Hospital Centre University of Nottingham Derby UK; ^2^ Department of Anaesthetics and Surgery Royal Derby Hospital Derby UK

**Keywords:** aging, exercise, fitness, health, high‐intensity interval training

## Abstract

**Objective:**

High‐intensity interval training (HIIT) has been shown to be more effective than moderate continuous aerobic exercise for improving cardiorespiratory fitness (CRF) in a limited time frame. However, the length of time required for HIIT to elicit clinically significant improvements in the CRF of older adults is currently unknown. The aim of this study was to compare changes in the CRF of older adults completing identical HIIT protocols of varying durations.

**Methods:**

Forty healthy, community‐dwelling older adults completed a cardiopulmonary exercise test (CPET) before and after 2, 4, or 6 weeks of fully supervised HIIT on a cycle ergometer, or a no‐intervention control period.

**Results:**

Anaerobic threshold (AT) was increased only after 4 (+1.9 [SD 1.1] mL/kg/min) and 6 weeks (+1.9 [SD 1.8] mL/kg/min) of HIIT (both *P* < 0.001), with 6‐week HIIT required to elicit improvements in VO_2_ peak (+3.0 [SD 6] mL/kg/min; *P* = 0.04). Exercise tolerance increased after 2 (+15 [SD 15] W), 4 (+17 [SD 11] W), and 6 weeks (+16 [SD 11] W) of HIIT (all *P* < 0.001), with no difference in increase between the groups. There were no changes in any parameter in the control group.

**Conclusion:**

Improvements in exercise tolerance from HIIT precede changes in CRF. Just 4 weeks of a well‐tolerated, reduced‐exertion HIIT protocol are required to produce significant changes in AT, with a further 2 weeks of training also eliciting improvements in VO_2_ peak.

## Graphical abstract

High intensity interval training (HIIT) is a time‐efficient exercise strategy to improve cardiorespiratory fitness. In older adults, 4‐weeks of our specific HIIT regime can improve anaerobic threshold while 6‐weeks is needed to improve VO_2_ peak.

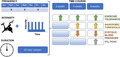

## 1 INTRODUCTION

The UK, like the majority of the Western world, has an aging population with those aged over 65 years projected to represent over a quarter of the population within the next 50 years.^1^ With this comes increasing prevalence of diseases associated with advancing age, many of which are treatable surgically.^2^ However, cardiorespiratory fitness (CRF) is known to decline with advancing age^3^ and is associated not only with increased all‐cause mortality,^4^ but also with a decreased ability to recover from a physiological insult (eg, surgery^5^). Thus, there remains a need to identify well‐tolerated exercise interventions to increase the CRF of older adults, with potential utility as a public health intervention^6^ or as “prehabilitation” prior to major surgery.^7^

Aerobic exercise training (AET) interventions are well‐known to be effective at improving a variety of health‐related variables in older adults, including CRF^8^ and resting blood pressure.^9^ However, one of the most commonly cited barriers to exercise adherence is “lack of time”^10^; this is especially true for AET, as sessions of this nature commonly last for 30 minutes or more, and in older adults may need 12 or more weeks to show benefit.^9^ A recent meta‐analysis has demonstrated that high‐intensity interval training (HIIT), with a significantly reduced time commitment, is more effective than moderate AET at improving CRF in older adults in less than 8 weeks.^11^

Despite numerous studies in the field of HIIT assessing the efficacy of different protocols for improving CRF^12^ and blood pressure,^13^ there is significant heterogeneity between the HIIT protocols used, with variations in intensity, duration, number of exertions, and recovery. Also, a large proportion of these studies are in young individuals, often utilizing supramaximal protocols (ie, Wingate) that would not be tolerable for the majority of older adults.^14^ Additionally, robust data on the time course of physiological adaptions to HIIT in older adults is lacking. One recent meta‐analysis did evaluate the effect of HIIT over different durations and demonstrated greater benefit from longer (>4 weeks) training durations; however, that analysis only included studies in younger adults, including athletic populations, and considered a variety of different HIIT regimes.^12^ Similarly, individual studies that have evaluated the time course of adaptations to a single HIIT protocol have so far only been conducted in younger population groups^15^ and we are unaware of any studies of this nature in older adults.

Based on a previously published HIIT protocol that was both well‐tolerated and effective at improving the CRF of older adults over a 4‐week period,^16^ the primary aim of this study was to determine the effect of either shortening or lengthening this training program on CRF adaptations in healthy older adults. We hypothesized that longer durations would lead to larger improvements, which may have clinical relevance given the mandated time constraints for interventions in certain age‐associated clinical conditions.^16^

## 2 MATERIALS AND METHODS

Institutional research ethics approval was obtained (J14112013‐A12092016) to recruit healthy, recreationally active (defined as not participating in a regular formal exercise), community‐dwelling volunteers aged 65‐85 years by local advertising. Thirty volunteers were assigned to participate in an identical HIIT program for either 2, 4, or 6 weeks, with 10 volunteers allocated to a non‐intervention control group. Before baseline assessments, all volunteers provided written informed consent and were examined by a qualified medical doctor with exclusion criteria for further participation taken from the American Thoracic Society (ATS)/American College of Chest Physicians (ACCP) guidelines for cardiopulmonary exercise testing (CPET).^17^

### 2.1 Pre‐ and post‐intervention testing

Testing began with a whole‐body dual‐energy X‐ray absorptiometry scan to assess body composition (Lunar Prodigy II, GE Medical Systems), providing automated data for whole body lean mass, body fat percentage, and lean leg mass.

Blood pressure was then measured in triplicate (mean value used) in the subject’s right arm using oscillometry (Datascope trio patient monitor, Datascope), with the subject in a seated position, after having rested for 5 minutes.^18^

A ramp incremental CPET was then performed according to the ATS/ACCP guidelines^17^ using a Lode Corival cycle ergometer (Lode Corival, Lode) and inline gas‐analysis system (ZAN 680, nSpire Health) as previously described^16^ to achieve a CPET duration of between 8 and 12 minutes.^17^ Anaerobic threshold (AT) was determined using a combination of the V‐slope and ventilatory‐equivalents methods^19^ by two blinded, experienced assessors with disagreement resolved by consensus. VO_2_ peak and exercise tolerance were determined as the oxygen utilization and power output (directly related to test duration) at volitional exhaustion, respectively. All baseline testing procedures were repeated 72 hours after the intervention period.

### 2.2 Interventions

Subjects assigned to a HIIT program attended our exercise laboratory three times each week for 2, 4, or 6 weeks to perform a previously published HIIT protocol.^16^ In brief, each training session lasted 16.5 minutes, comprising a 2‐minute warm‐up of unloaded cycling followed by five 1‐minute intervals at 90%‐110% of the peak power output achieved during the pre‐intervention CPET. Working intervals were interspersed with 90 seconds of active recovery cycling, with a final 3.5‐minute active recovery and monitoring period. Each training session was fully supervised with continuous 12‐lead electrocardiogram monitoring throughout. Subjects in the control group attended only for pre‐ and post‐intervention testing, 6 weeks apart. All subjects were asked to maintain their habitual diet and levels of physical activity for the duration of the study.

### 2.3 Statistics

All calculations were performed using GraphPad Prism Version 7.02. Data are presented as mean (SD). Data were tested for normality and analyzed appropriately. Participant demographics and outcome variables at baseline were compared using one‐way analysis of variance (ANOVA), whilst outcome data were compared using two‐way ANOVA for time (pre‐/post‐intervention) and group with Sidak’s post hoc testing. Significance was taken as an alpha of *P* < 0.05.

## 3 RESULTS

### 3.1 Subject characteristics

Forty subjects, mean age (SD) 71 (5) years, mean BMI (SD) 25.8 (2.6) kg/m^2^, were recruited into this study. There were no significant differences in sex, age, baseline height or weight (Table 1), or in any outcome variable (all *P* > 0.1) between the groups at baseline. All 40 participants completed the study with none lost to follow‐up. Training compliance was 100% with no adverse events reported.

**TABLE 1:** Subject characteristics

Group	Age, y	Weight, kg	Height, m	Male/Female, n
Control	73 (6)	74 (7)	1.7 (0.1)	6/4
2‐Wk HIIT	73 (4)	69 (9)	1.7 (0.1)	5/5
4‐Wk HIIT	68 (3)	71 (10)	1.7 (0.1)	5/5
6‐Wk HIIT	69 (3)	80 (13)	1.7 (0.1)	5/5

Note

Data are presented as mean (SD). There were no significant differences between the groups.

Abbreviation: HIIT, high‐intensity interval training.

### 3.2 Anaerobic threshold

**Figure 1:**
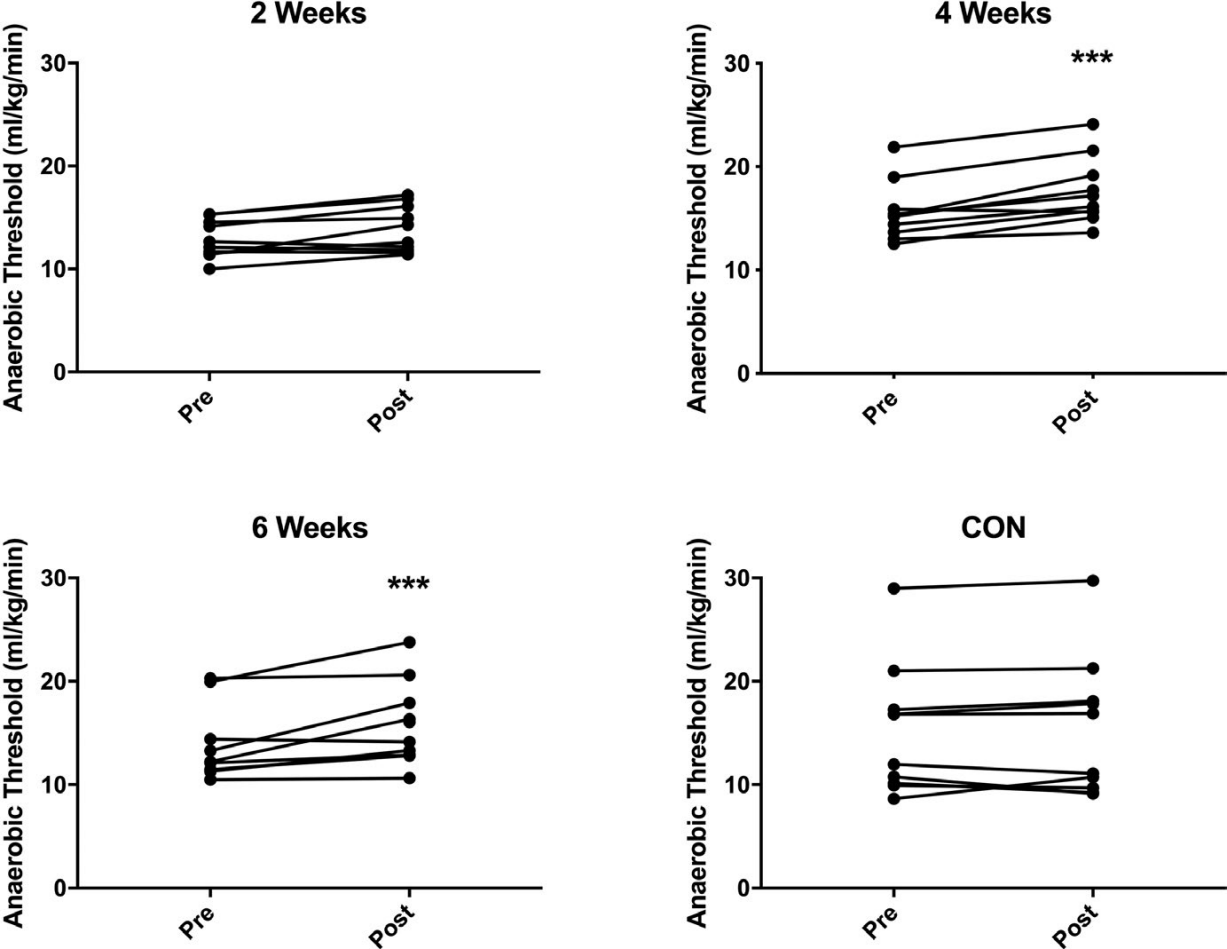
Anaerobic threshold before (pre) and after (post) 2‐wk, 4‐wk, or 6‐wk high‐intensity interval training (HIIT), or a no‐intervention control period. N = 10 subjects per group. Analysis via two‐way analysis of variance with Sidak’s multiple comparison post hoc analysis. ***P < 0.001 vs pre‐HIIT.

There was a main effect of time across groups (*P* < 0.001) and a significant Group × Time interaction (*P* = 0.01). Post hoc analysis demonstrated a significant increase in AT in the 4‐week (15.6 ± 2.8 vs 17.6 ± 3.2 mL/kg/min; *P* <0.001) and 6‐week (14.0 ± 3.7 vs 15.8 ± 4.3 mL/kg/min; *P* = <0.001) groups after HIIT, with no significant change in either the 2‐week (12.9 ± 1.8 vs 13.9 ± 2.3 mL/kg/min; *P* = 0.08) or control groups (15.2 ± 6.3 vs 15.4 ± 6.7 mL/kg/min; *P* = 1.00; Figure 1).

### 3.3 VO_2_ peak

**Figure 2:**
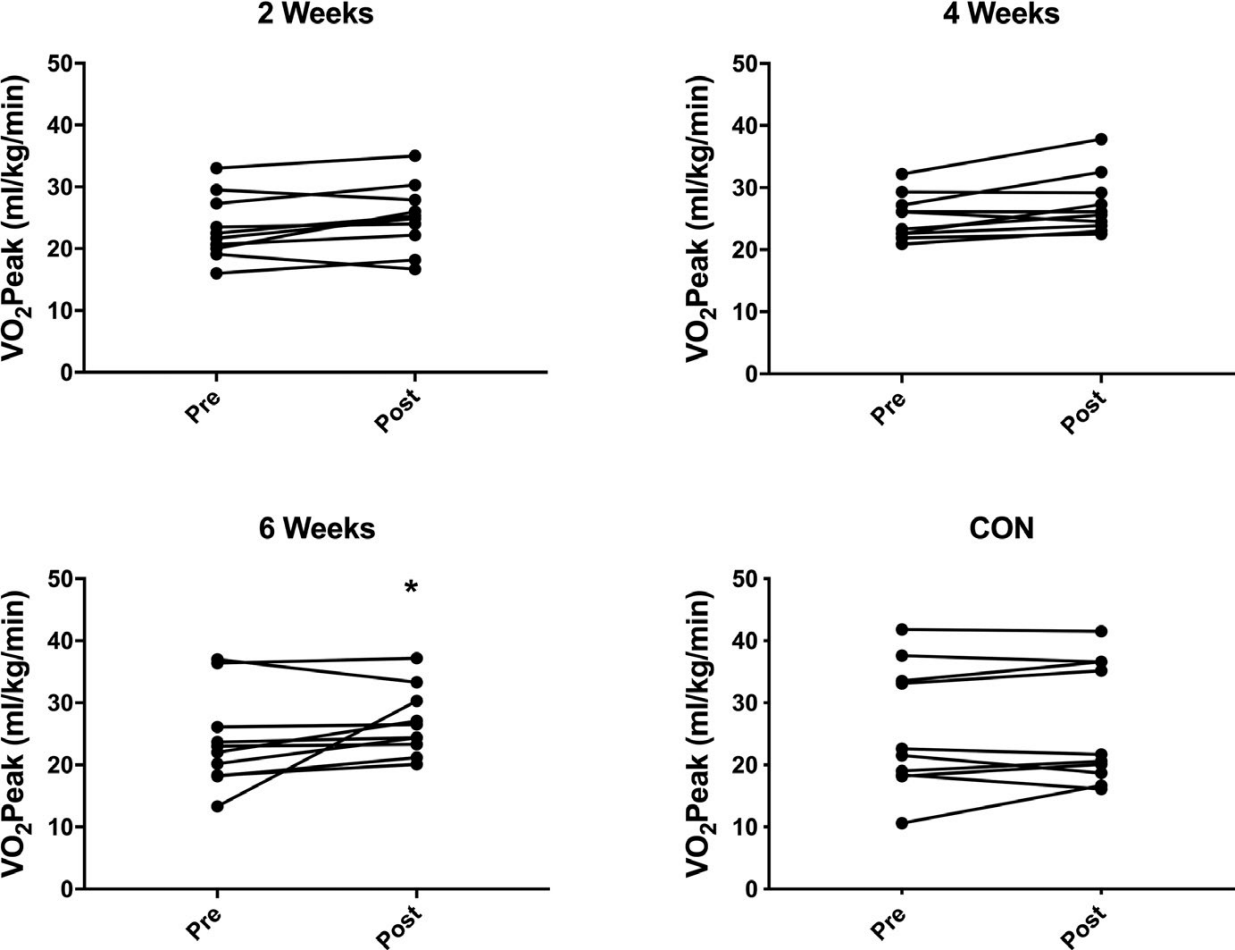
Peak oxygen consumption before (pre) and after (post) 2‐wk, 4‐wk, or 6‐wk high‐intensity interval training (HIIT), or a no‐intervention control period. N = 10 subjects per group. Analysis via two‐way analysis of variance with Sidak’s multiple comparison post hoc analysis. *P < 0.05 vs pre‐HIIT.

There was a main effect of time across groups (*P* < 0.002), but no significant Group × Time interaction. Post hoc analysis demonstrated a significant increase in VO_2_ peak only in the 6‐week group (23.8 ± 7.7 vs 26.8 ± 5.4 mL/kg/min; *P* = 0.046; Figure 2), with no significant change after 2 weeks (23.3 ± 5.2 vs 25.1 ± 5.4 mL/kg/min; *P* = 0.43) or 4 weeks (25.2 ± 3.6 vs 27.2 ± 4.8 mL/kg/min; *P* = 0.28) of HIIT, or in the control group (25.6 ± 10.1 vs 26.4 ± 9.8 mL/kg/min; *P* = 0.94; Figure 2).

### 3.4 Exercise tolerance

**Figure 3:**
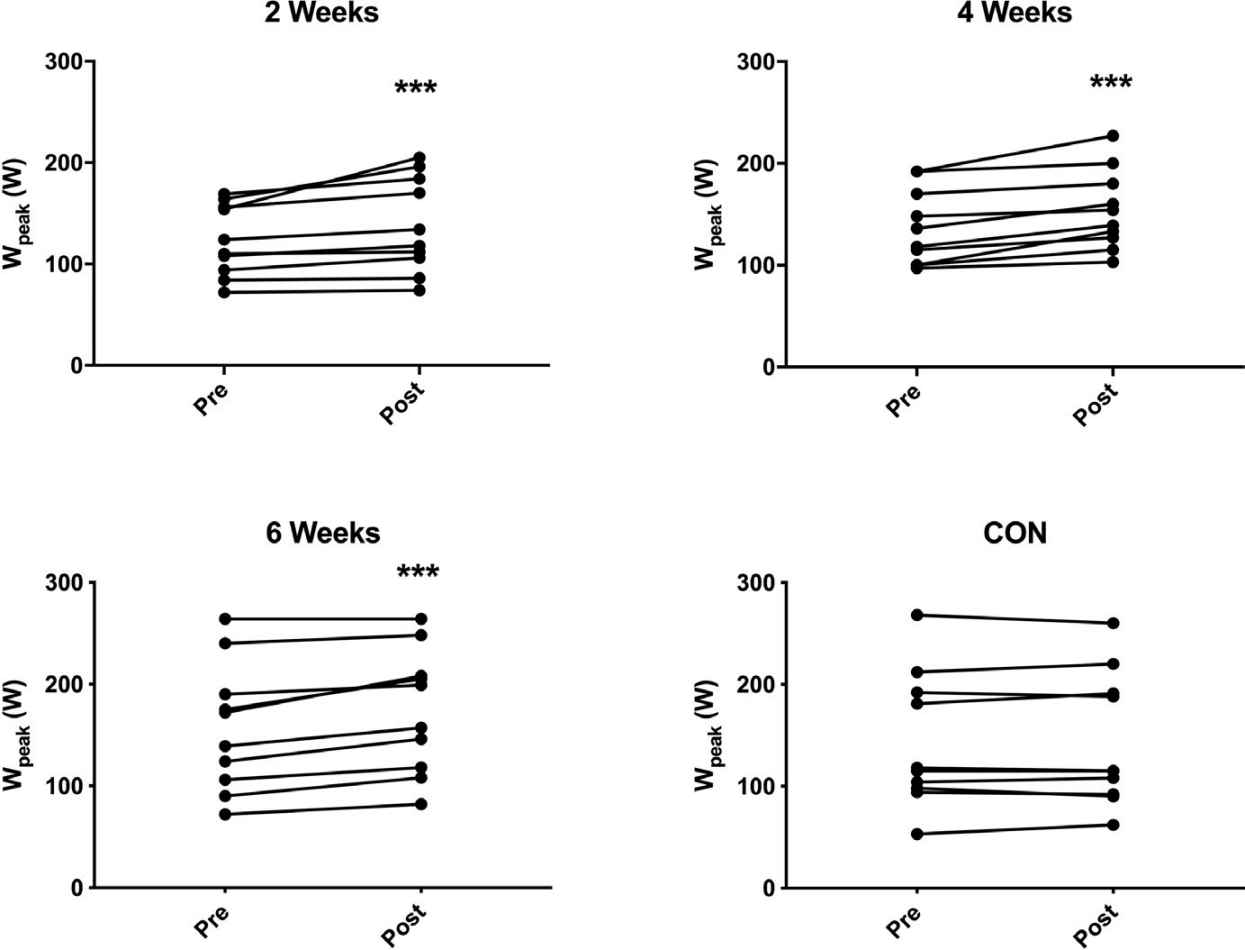
Peak power output before (pre) and after (post) 2‐wk, 4‐wk, or 6‐wk high‐intensity interval training (HIIT), or a no intervention control period. N = 10 subjects per group. Analysis via two‐way analysis of variance with Sidak’s multiple comparison post hoc analysis. ***P < 0.001 vs pre‐HIIT.

For exercise tolerance, determined by peak power at termination of CPET (which is directly related to CPET duration), there was a main effect of time across groups (*P* < 0.001) and a significant Group × Time interaction (*P* = 0.007). Post hoc analysis demonstrated a significant increase in the 2‐week (124 ± 35 vs 139 ± 47 W), 4‐week (137 ± 37 vs 154 ± 39 W), and 6‐week (157 ± 63 vs 174 ± 61 W) HIIT groups (all *P* < 0.001), with no significant change in the control group (144 ± 66 vs 144 ± 66 W; *P* = 1.0; Figure 3).

### 3.5 Blood pressure

For resting systolic blood pressure, there was a main effect of time across groups (*P* < 0.003), but no significant Group × Time interaction. Post hoc analysis demonstrated a significant decrease in resting systolic blood pressure in both the 4‐week (140 ± 19 vs 132 ± 18 mmHg; *P* = 0.047) and 6‐week (143 ± 18 vs 134 ± 12 mmHg; *P* = 0.02) HIIT groups, with no significant change in either the 2‐week HIIT (131 ± 12 vs 129 ± 17 mmHg; *P* = 0.90) or control groups (128 ± 10 vs 128 ± 11 mmHg; *P* = 1.00). There was no significant change in diastolic blood pressure in any group (2 weeks: 79 ± 9 vs 78 ± 9 mmHg; *P* = 0.99; 4 weeks: 82 ± 7 vs 74 ± 12 mmHg; *P* = 0.14; 6 weeks: 86 ± 14 vs 83 ± 8 mmHg; *P* = 0.83; control: 79 ± 10 vs 77 ± 7 mmHg; *P* = 0.97) with no main effect of time across groups, nor significant Group × Time interaction.

### 3.6 Body composition

There was no significant change in any of our body composition parameters (whole body lean mass, body fat percentage, and lean leg mass) in any group (Table 2), with no main effect of time across groups, nor significant Group × Time interaction.

**TABLE 2:** Body composition parameters before and after 2‐, 4‐, or 6‐wk HIIT, or a no intervention control period

Group	Before HIIT	After HIIT	Change	*P* value
Control LM (Kg)	49.9 (5.4)	49.9 (5.0)	+0.0 (1.2)	1.0
2‐wk LM(Kg)	43.9 (8.6)	44.5 (2.8)	+0.5 (0.7)	0.36
4‐wk LM(Kg)	44.6 (10.9)	44.7 (10.8)	+0.1 (1.1)	1.0
6‐wk LM(Kg)	48.3 (11.2)	48.4 (11.3)	+0.2 (0.8)	0.98
Control FM%	29.5 (7.3)	29.5 (6.8)	0.0	1.0
2‐wk FM%	32.7 (7.1)	32.2 (7.0)	−0.5 (0.9)	0.55
4‐wk FM%	32.0 (10.1)	31.7 (9.6)	+1.7 (0.6)	0.91
6‐wk FM%	38.0 (6.3)	37.4 (6.2)	−0.5 (0.7)	0.43
Control LLM (Kg)	15.5 (4.1)	15.7 (4.4)	+0.2 (1.6)	1.0
2‐wk LLM (Kg)	14.3 (3.1)	14.6 (3.1)	+0.3 (0.4)	0.97
4‐wk LLM (Kg)	14.6 (4.2)	14.4 (4.3)	−0.2 (0.6)	1.0
6‐wk LLM (Kg)	13.4 (2.0)	14.0 (3.4)	+0.7 (2.7)	0.63

Note

Analysis via dual‐energy x‐ray absorptiometry. Data are presented as mean(SD). *P* values represent change from before HIIT to after HIIT via two‐way repeated‐measures analysis of variance with Sidak’s multiple comparisons post hoc testing.

Abbreviations: FM%, whole‐body fat percentage; HIIT, high‐intensity interval training; LLM, leg lean mass; LM, whole body lean mass.

## 4 DISCUSSION

This study has demonstrated that a time‐efficient, well‐tolerated HIIT program elicits appreciable improvements in exercise tolerance before any measurable changes in CRF in a cohort of older adults. Two weeks of HIIT appears to be insufficient to improve AT, but this can be achieved with 4 or 6 weeks of training. Six weeks of HIIT is seemingly required to improve VO_2_ peak. To our knowledge, this is the first study to attempt to plot the time course of CRF changes resulting from a single HIIT protocol in healthy older adults.

Supporting the efficacy of our specific HIIT protocol, other studies of HIIT in older adults have demonstrated improvements in VO_2_ peak of a similar magnitude to ours after 6 and 8 weeks of HIIT,^20^ with longer‐duration studies (12 weeks) demonstrating gains approaching 5 mL/kg/min.^21^ Likewise for changes in AT, similar improvements to ours have been seen at 6 weeks,^22^ with longer studies demonstrating improvements of ~4 mL/kg/min at 12 weeks.^21^ However, these data are drawn from different HIIT protocols and not different durations of a single HIIT protocol and as such cannot be used to truly determine the temporal nature of adaptations to HIIT in older adults.

The physiological basis behind our observation that significant improvements in AT occur before increases in VO_2_ peak may reside at the mitochondrial level. Mitochondrial biogenesis pathways, arguably the main determinant of AT, are seen to be upregulated after just a single HIIT session^23^ and therefore could be important early in a training regime. Conversely, central cardiovascular adaptions (ie, blood volume and cardiac output), which are thought to be the main mechanisms through which HIIT may influence VO_2_ peak, tend to occur later in a training programme.^24^ Although we were unable to find any previous studies evaluating HIIT over durations as short as 2 weeks in older adults, those conducted in young men showed similar findings to ours: an increase in peak power without significant changes in CRF.^25^ It may therefore be that the peak power increases seen very early in training are a consequence of improved cycling efficiency and improved exercise tolerance,^25^ after which changes in AT related to mitochondrial upregulation become apparent before, finally, central cardiovascular changes manifest.

Despite our HIIT program eliciting CRF and blood pressure improvements in just 4 weeks and physical function gains in just 2 weeks, it did not lead to any significant changes in body composition over 2, 4, or 6 weeks. This is despite previous studies in older adults demonstrating gains in lean mass^26^ and reductions in body fat^27^ with HIIT. This disparity may be due to differences in participant demographics and/or training modalities. For example, Herbert and colleagues^26^ employed 30‐second Wingate sprints in an exclusively male cohort of participants who were a decade younger than our cohort, including some masters’ athletes, and who were able to demonstrate a ~3% increase in lean mass with 6 weeks of HIIT. Søgaard and colleagues^27^ also studied a cohort of participants who were a decade younger than our participants and who were overweight or obese and failed to significantly increase in lean mass, but were able to significantly reduce their body fat percentage by ~0.6%. In support of the notion of exercise modality having a role to play in disparate body composition changes in response to HIIT, a recent systematic review of previous studies (albeit based on studies largely in younger adults) has demonstrated cycling‐based HIIT to be far less effective than running HIIT for reducing body fat percentage,^28^ likely due to the recruitment of more muscle groups when running.

One final consideration as to the reason for the lack of body composition changes must be given to diet, which was not controlled or measured in this study, although participants were instructed to maintain their habitual pre‐study dietary behavior. There is a possibility that our HIIT may have led to an increase in appetite and subsequent energy intake that may have diminished body fat reductions.

This study has also demonstrated significant reductions in resting systolic blood pressure with just 4 weeks of HIIT. This reduction of 8 mmHg systolic blood pressure is greater than that previously observed in trials of both moderate AET and resistance exercise training in older adults,^9^ but is similar to that observed in the few previous studies of HIIT in older adults that have measured this.^22^ Of interest, the 4 weeks that our HIIT protocol required to produce significant changes in blood pressure is much shorter than that described in the majority of exercise trials in older adults, with the majority lasting for 3 months or longer.^9^

One important feature of the training program used herein is its tolerability, as was demonstrated by the 100% training compliance in a cohort with a mean age over 70 years. This demonstrates its suitability for use in older adults per se and in older patient groups, where adherence to previous exercise prehabilitation has been reported to be as low as 16%.^29^

That our HIIT protocol can increase AT in 4 weeks would allow this protocol to be used as exercise prehabilitation before, for example, major surgical intervention for cancer where there is a limitation on the preoperative time available: for example, the 31‐day time‐to‐first‐treatment cancer target in the UK.^30^ An increase in CRF prior to major surgery has been shown to reduce both perioperative morbidity and mortality,^5^ with ongoing large randomized clinical trials of exercise prehabilitation (ie, NCT03509428) currently underway to fully determine the effects of exercise prehabilitation on direct clinical outcomes (eg, complication rates and length of hospital stay). Although 6 weeks of HIIT appears to be needed to improve VO_2_ peak, this measure is prone to a substantial degree of subjectivity with participant effort a significant contributing factor. As such, our data would not support a delay to any preexisting clinical treatment targets beyond 4 weeks to achieve this.

As with all studies, we must acknowledge some limitations to our study design. That we did not have a single intervention group with repeated measurements at baseline, 2, 4, and 6 weeks is our main limitation. However, incorporating repeated CPET assessments every 2 weeks would have impacted delivery of our HIIT protocol (as these sessions would have had to replace HIIT sessions) and based on patient and public involvement feedback from our participants, was not something many of them would have been willing to undertake. We also acknowledge that prescribed exercise interventions interact with habitual physical activity and nutritional intake, aspects that were not measured in this study. We did, however, ask all participants to maintain their normal diet and levels of physical activity for the duration of the study.

In conclusion, this study is the first to map the time course of physiological adaptions to a single, well‐tolerated HIIT protocol in older adults, demonstrating that a 5‐by‐1‐minute HIIT protocol performed 3‐times each week can elicit significant improvements in the exercise tolerance of older adults in just 2 weeks, whilst 4 weeks of this training regime can lead to significant improvements in CRF and resting systolic blood pressure.

## CONFLICTS OF INTEREST

Nothing to disclose.

## AUTHOR CONTRIBUTION

P.J.J.H., J.E.M.B., and B.E.P. conceived and designed the study. P.J.J.H. and B.E.P. co‐wrote the manuscript. P.J.J.H., J.E.M.B., and C.L.B. performed the human studies, acquired the data, and performed data analysis. P.J.J.H., J.E.M.B., C.L.B., P.J.A., J.N.L., J.P.W., and B.E.P. read and approved the final manuscript.
